# A Case Report Demonstrating How the Clinical Presentation of the Diffuse Sclerosing Variant of Papillary Thyroid Carcinoma Can Mimic Benign Riedel's Thyroiditis

**DOI:** 10.1155/2015/686085

**Published:** 2015-06-02

**Authors:** Jennifer Walsh, Tomas P. Griffin, Carmel B. Ryan, James Fitzgibbon, Patrick Sheahan, Matthew S. Murphy

**Affiliations:** ^1^Department of Endocrinology, South Infirmary Victoria University Hospital, Cork, Ireland; ^2^Department of Pathology, Cork University Hospital, Cork, Ireland; ^3^Department of Otolaryngology-Head and Neck Surgery, South Infirmary Victoria University Hospital, Cork, Ireland

## Abstract

A 44-year-old female presented with a two-month history of a neck mass, sore throat, hoarseness, and intermittent dysphagia. Examination revealed a “woody” hard swelling arising from the right lobe of the thyroid. Clinically this was felt to be classical Riedel's thyroiditis (RT). Thyroid ultrasound showed a diffusely enlarged, low echogenicity thyroid with a multinodular goitre. An abnormal nodule extending across the isthmus was noted. Following a nondiagnostic fine needle aspiration, an open core biopsy was performed. This showed dense sclerotic fibrosis punctuated by nodular mononuclear inflammatory cells, which obscured follicular epithelial cells consistent with a fibrosing thyroiditis (Riedel's thyroiditis). A biopsy of pretracheal lymph nodes showed a sclerotic process throughout the lymph nodes and nests of epithelium bands with squamous differentiation obscured by a fibrous process. These findings raised the differential diagnosis of diffuse sclerosing variant of papillary thyroid carcinoma (DSV-PTC) with metastasis to lymph nodes. A total thyroidectomy and pretracheal lymph node dissection were performed. The final histological diagnosis was DSV-PTC. When managing a patient with presumed RT it is important to consider malignancy in the differential. DSV-PTC is one of the more aggressive forms of thyroid cancer but with early diagnosis and appropriate treatment patients may have excellent outcomes.

## 1. Introduction

Riedel's thyroiditis is a rare benign inflammatory process involving the thyroid gland and surrounding cervical tissue [1], first described by Riedel in 1896 [2]. Diffuse sclerosing variant of papillary thyroid carcinoma (DSV-PTC) is a rare aggressive form of papillary thyroid carcinoma [3]. DSV-PTC has been described as a “wolf in sheep's clothing” [4] and presentation is often indicative of benign disease [5, 6]. We describe the case of a lady whose clinical presentation was suggestive of benign Riedel's thyroiditis but following extensive investigation was shown to have DSV-PTC. As this condition has a good prognosis when identified and treated early, a high index of suspicion must be maintained in all cases of suspected Riedel's thyroiditis.

## 2. Case

A 44-year-old female presented with a two-month history of a neck mass, sore throat, hoarseness, and intermittent dysphagia. Examination revealed a “woody” hard swelling arising from the right lobe of the thyroid gland with no cervical lymphadenopathy. Clinically this was felt to be classical Riedel's thyroiditis (RT). Biochemically, the patient was hypothyroid (T_4_ 0.8 pmol/L; TSH > 100 mIU/L). Anti-thyroid peroxidase antibodies were >1000 units/mL. Flexible laryngoscopy demonstrated bilateral Reinke's oedema of the vocal cords with no suspicious lesions identified. Thyroid ultrasound showed a diffusely enlarged, low echogenicity thyroid gland, heterogenous in echotexture with evidence of a multinodular goitre. No calcification was noted. An abnormal nodule (4 cm by 1.5 cm) extending across the isthmus was noted. Magnetic resonance imaging revealed diffuse enlargement of the entire thyroid gland with marked enhancement following administration of contrast. No focal nodules were identified. Several prominent jugular nodes were seen bilaterally, benign in size and appearing reactive in nature.

Following a nondiagnostic fine needle aspiration, an open core biopsy of the thyroid nodule was performed. This showed dense sclerotic fibrosis punctuated by nodular mononuclear inflammatory cells, which obscured follicular epithelial cells consistent with a fibrosing thyroiditis (Riedel's thyroiditis); differential diagnosis included fibrous variant of Hashimoto's thyroiditis. However, a biopsy of pretracheal lymph nodes performed at the same time showed a sclerotic process throughout the lymph nodes and nests of epithelium bands with squamous differentiation obscured by a fibrous process. These findings raised the differential diagnosis of DSV-PTC with metastasis to lymph nodes.

Based on lymph node biopsy, nodule size, sudden onset of a neck mass, and cosmetic appearance, a total thyroidectomy and pretracheal lymph node dissection were performed. At the time of surgery, the thyroid was found to be diffusely enlarged, extremely indurated, and difficult to mobilize with no gross evidence of extra thyroidal spread of tumour and no suspicious lymph node enlargement.

**Figure 1:**
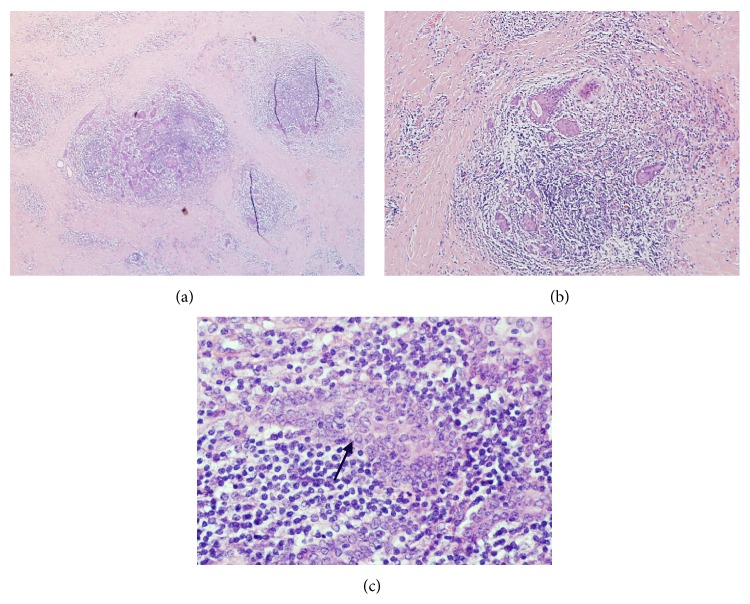
(a) Medium magnification: obliteration of architecture by dense fibrosis. (b) High magnification: chronic inflammatory infiltrate, squamous metaplasia, and nuclear features of papillary thyroid carcinoma. (c) Higher magnification: papillary thyroid cancer cells with typical nuclear changes (indicated by arrow).

The final histological diagnosis was DSV-PTC (T3N1a) based on the characteristic findings as shown in Figures 1(a), 1(b), and 1(c).

## 3. Discussion

RT is a rare fibrotic condition that results in destruction of the thyroid gland and infiltration of the surrounding tissue [1]. The estimated incidence is 1.06 per 100,000 affecting women up to 3 times more commonly than men [7]. Like in our case, patients present with a “woody” hard thyroid gland and often have compressive and infiltrative symptoms such as dyspnoea, dysphagia, and hoarseness. Hypothyroidism can occur due to replacement of the thyroid parenchyma with fibrous tissue [1]. Definitive diagnosis is based on histological criteria: fibroinflammatory process involving all or a portion of the thyroid gland, evidence of extension into surrounding tissues, infiltrates of inflammatory cells without giant cells, lymphoid follicles, oncocytes or granulomas, evidence of occlusive phlebitis, and absence of malignancy [7].

PTC has a higher incidence (5.6 per 100,000 person-years) than RT [8]. Like Riedel's thyroiditis, it is more common in women (8.8 per 100,00 woman-years versus 2.7 per 100,000 man-years) [8]. DSV-PTC is uncommon accounting for 1.8% of all PTCs [3]. Patients with DSV-PTC present at a younger age and larger mean size of tumour with a higher incidence of cervical node metastasis compared to patients with classical PTC [3]. Presenting features can be similar to RT. Diagnosis is based on the presence of several pathological features including a diffuse firm enlargement of the thyroid gland with scattered islands of papillary carcinoma, extensive lymphatic permeation and lymphocytic infiltration, sclerosis, squamous metaplasia, and psammoma bodies [9]; it is these features which are essential in distinguishing PTC from benign pathologies such as Riedel's thyroiditis.

When managing a patient with presumed RT it is important to consider malignancy in the differential as patients can present with similar symptoms. If any concern regarding malignancy exists, patients need to undergo further investigations, until malignancy is definitively excluded. DSV-PTC is one of the more aggressive forms of thyroid cancer but with early diagnosis and appropriate treatment patients may have excellent outcomes (5-year survival >90%) [10].
